# Reaction time variations in normal aging and elderly MCI patients under various cognitive load conditions

**DOI:** 10.3389/fnhum.2025.1623252

**Published:** 2025-07-23

**Authors:** Yanfei Zhu, Zhuoming Chen, Zhengkun Shi, Junyi Chen, Qiankun Zuo, Zhi Yang, Wenjing Zhang, Wanting Li, Siyu Lu, Siyuan Peng, Lei Gou

**Affiliations:** ^1^College of Education Science, Hubei Normal University, Huangshi, China; ^2^School of Foreign Languages, Sun Yat-sen University, Guangzhou, China; ^3^Department of Rehabilitation Medicine, The First Affiliated Hospital of Jinan University, Guangzhou, China; ^4^School of Information Engineering, Hubei University of Economics, Wuhan, Hubei, China; ^5^Hubei Key Laboratory of Digital Finance Innovation, Hubei University of Economics, Wuhan, Hubei, China; ^6^College of Electronics and Information Engineering, Sichuan University, Chengdu, China; ^7^Department of Rehabilitation Medicine, Shenzhen Maternity and child Healthcare Hospital, Shenzhen, China; ^8^Department of Rehabilitation Medicine, Jiangmen Central Hospital, Jiangmen, China; ^9^Department of Rehabilitation Medicine, Maoming People's Hospital, Maoming, China; ^10^Department of Rehabilitation Medicine, The Fifth Affiliated Hospital of Jinan University, Heyuan, China

**Keywords:** mild cognitive impairment, stroop effect, cognitive processing, reaction time, interference control, cognitive assessment, neuropsychological testing

## Abstract

**Objectives:**

To compare reaction time parameters and accuracy rates between cognitively normal older adults and those with mild cognitive impairment (MCI) during the Stroop Color-Word Test, and to investigate how cognitive load modulates performance in MCI.

**Methods:**

Speech audio samples (*n* = 1,920) were collected from 10 cognitively normal older adults and 10 MCI patients during Stroop task execution. Accuracy and reaction time were extracted. Analysis of variance and multiple comparison were used to analyze the differences in reaction time under different task conditions within the group, while the independent sample t-test was used to compare the accuracy and reaction time of the two groups under the same task. Pearson correlation analysis was used to determine the linear relationship between MOCA scores and the accuracy rate and reaction time of MCI patients in the interference suppression task.

**Results:**

The accuracy rate of the mild cognitive impairment (MCI) group was significantly lower than that of the control group (*p* < 0.05). Tasks A-D had different effects on reaction times, with significant main effects observed in both the NC group (*P* = 0.000, η^2^ = 0.637) and the MCI group (*P* = 0.000, η^2^ = 0.721). Reaction times in both groups prolonged with increasing cognitive load (*p* < 0.05), but the delay was more pronounced in the MCI group (p < 0.05). A positive linear correlation was found between the MoCA score and task accuracy rate (*r* = 0.758, *P* = 0.011).

**Conclusion:**

Dominant responses require less processing time, whereas tasks demanding interference suppression elicit slower reaction times and higher error rates. MCI patients demonstrate prolonged reaction times and greater susceptibility to proactive interference compared to controls, highlighting impaired interference control mechanisms. These findings suggest that MCI is characterized by early deficits in dominance inhibition, manifesting as reduced ability to suppress automatic responses and increased vulnerability to cognitive conflict.

## 1 Introduction

Mild cognitive impairment (MCI) represents a critical transitional stage between normal aging and dementia. It is defined by mild declines in cognitive function, which may affect single or multiple domains such as memory, language, and attention (Farias et al., 2006; Lenzi et al., 2011). Individuals with MCI often self-report these cognitive abnormalities, and standardized neuropsychological assessments can objectively validate the presence of objective cognitive impairment–though its severity remains below the diagnostic threshold for dementia (Jacova et al., 2007; Belleville et al., 2017). A core feature of MCI is the coexistence of subjective and objective cognitive decline. It is also highly heterogeneous, typically classified into single-domain or multi-domain subtypes. MCI follows a dynamically reversible disease course: some cases may stabilize or even reverse with addressing treatable factors, whereas approximately 10%–15% of individuals progress to dementia annually, carrying a risk 5–10 times higher than the general population (Solfrizzi et al., 2004; Davis et al., 2018). At the individual level, MCI is associated with reduced quality of life, frequent occurrences of anxiety and depression, and a significantly elevated risk of developing dementia. 5-year conversion rate of amnestic MCI to dementia is reported to be around 50% (Mitchell and Shiri-Feshki, 2008; Rountree et al., 2007). Early identification of MCI enables timely interventions–such as cognitive training and exercise programs–that can effectively delay disease progression (Zuo et al., 2024; Fowler et al., 2025; Zuo et al., 2025b).

MCI detection have been widely explored using machine learning models. These models are developing along the pathogenic mechanisms of cognitive functions, such as lesions in the affected brain regions and abnormal connections between brain regions (Zuo et al., 2025c; Zhu et al., 2024; Zuo et al., 2025a). The direct way to explore these pathogenic mechanisms is to assess the abnormalities of the human body's perceptual functions, including memory, language, orientation, praxis, attention, and executive function (Zhuang et al., 2021). Numerous assessment tools have been developed to detect global and domain-specific cognitive impairments, frequently employed in both clinical and research contexts to aid in the diagnosis of Alzheimer's disease (AD) and identification of MCI (Huo et al., 2021; Gutierrez et al., 2021). However, these commonly used cognitive screening instruments may fail to reliably detect subtle impairments in individuals with MCI. In contrast, language assessments have demonstrated greater sensitivity to early cognitive changes (Robin et al., 2021). In mild cognitive impairment patients, vocabulary deficits often manifest as increased pronoun usage and reduced noun frequency. Individuals tend to substitute pronouns for specific target nouns that are difficult to retrieve, while maintaining relatively fluent spontaneous speech despite underlying semantic retrieval challenges. Verbal memory tasks–such as word recall or narrative recall–have shown superior diagnostic utility in identifying MCI (Mueller et al., 2022). Although language performance is not the sole criterion for diagnosing MCI, substantial evidence highlights its significant role (McCullough et al., 2019; Sanderson-Cimino et al., 2022). Almor et al. (1999) proposed the working memory impairment hypothesis, suggesting that increased pronoun use in MCI is more closely linked to working memory deficits than to the severity of semantic degradation itself. Vuorinen et al. (2000) analyzed discourse from 48 individuals with MCI or mild-to-moderate AD and found that MCI participants produced fewer semantic units than controls. Tomoeda and Bayles (1993) further confirmed that reduced semantic content serves as a sensitive marker of AD progression, particularly valuable for early MCI screening. Speech fluency–the ability to produce fluid and coherent speech–is a fundamental measure of language competence. Themistocleous et al. (2020) analyzed speech samples from 26 MCI patients and demonstrated that fluency metrics were highly sensitive in differentiating MCI from healthy controls. Speech fluency is typically categorized into two subtypes: phonemic fluency (also termed letter fluency or verbal fluency), which reflects the ability to generate words starting with a specific letter or sound, and semantic fluency, which involves producing words within a specific category. In early-stage AD, word-finding difficulties and repetitions often compromise fluency. While both phonemic and semantic fluency are affected in early AD, semantic fluency is generally more sensitive to MCI detection. McDonnell et al. (2020) utilized semantic fluency as a screening tool in a community outpatient cohort of 232 older adults and found it significantly improved MCI detection sensitivity (81.2%) and specificity (78.8%) compared to the MMSE alone.

Current research has explored MCI-related language features across the different domains. Temporal characteristics of spontaneous speech are recognized as critical acoustic markers for differentiating individuals with MCI from healthy controls. Tóth et al. (2018) demonstrated that four temporal parameters–articulation clarity, speech rate, pause rate, and grammatical error rate–effectively distinguish MCI patients from cognitively normal individuals, with these metrics also correlating with the severity of cognitive impairment. Similarly, Singh et al. (2001) manually extracted and quantified temporal speech features in MCI patients but highlighted the limitations of time-consuming manual methods, which yield only approximate estimates of parameters such as speech rate and clarity. Working memory or semantic memory impairments in MCI may constrain the production of grammatically complex structures. Cross-linguistic studies have shown that MCI patients exhibit significantly reduced use of complex syntactic constructions compared to healthy controls (Sung et al., 2020), suggesting that syntactic simplification emerges during the early stages of cognitive decline. However, some researchers contend that syntactic structure remains relatively preserved during the prodromal phase of AD. Ahmed et al. (2012) observed group-level differences in global measures such as mean length of utterance (MLU) and grammatical error rates between MCI patients and controls, though individual syntactic variables did not consistently reach statistical significance. Roark et al. (2011) similarly found stable MLU in MCI but noted marked declines in syntactic complexity among patients with moderate-to-severe AD. Despite inconsistent findings, a consensus exists that syntactic simplification is observable in individuals with MCI. Researchers have proposed that fluctuations in cognitive load induce changes in respiratory and phonatory function, which manifest in the acoustic properties of speech. Özseven and Düğenci (2018) utilized Praat software to conduct spectral analysis of speech samples, focusing on temporal and acoustic features. Key variables–including percentage of voice breaks, number of voice periods, interruption frequency, shimmer (amplitude perturbation), and noise-to-harmonics ratio–were found to reliably distinguish AD patients from healthy controls. López-de Ipiña et al. (2015) further refined AD differentiation by incorporating features from the time domain, spectral domain, and fractal dimension of voice signals.

The Stroop task has become one of the most widely employed paradigms in cognitive psychology for eliciting interference effects. Numerous studies have integrated the Stroop Color-Word Test (CWT) into clinical research, demonstrating its utility in identifying individuals with mild dementia (Lin and Lai, 2024; Rao et al., 2025). Neuroimaging research has deepened our understanding of the neural mechanisms underlying the Stroop effect (Herd et al., 2006; Huang et al., 2020). During Stroop task performance, consistent activation has been observed in the inferior frontal gyrus (IFG), middle frontal gyrus (MFG), and dorsolateral prefrontal cortex (DLPFC) of both hemispheres (Jalalvandi et al., 2020). Evidence suggests the left IFG plays a particularly critical role in classic Stroop tasks involving written or spoken language stimuli, owing to its central function in language processing (Heidlmayr et al., 2020). Liu et al. (2006) used functional magnetic resonance imaging (fMRI) to show that distinct prefrontal cortex regions mediate response-related and non-response-related attentional control during the Stroop Color-Word Task, indicating functional specialization in executive control processes within the prefrontal cortex. Specifically, the dorsal left DLPFC was linked to supporting non-dominant response selection. Yeung et al. employed functional near-infrared spectroscopy (fNIRS) to demonstrate developmental variations in prefrontal activation during Stroop interference inhibition (Yeung et al., 2020). Similarly, Xiang et al. used fNIRS in a cross-sectional study and found that attentional conflict during Stroop tasks significantly modulated prefrontal activation (Xiang et al., 2023). These findings collectively enhance our understanding of the relationship between cognitive control and localized brain activity. Informed by these observations, the present study aims to investigate differences in reaction times and accuracy rates during cognitive processing between cognitively normal older adults and those with cognitive impairment. Using the Stroop Color-Word Test, we examine how cognitive load influences response performance in individuals with cognitive dysfunction. By analyzing dynamic changes in cognitive reaction times, this research seeks to provide novel insights for the detection and treatment of MCI.

## 2 Method

### 2.1 Study data

Participants were recruited from the First Affiliated Hospital of Jinan University and the university's community health service via public outreach campaigns raising awareness of cognitive impairment and the study's objectives. Initially, 23 older adults were enrolled. After excluding two individuals due to dialect-related interference and one due to voluntary withdrawal, valid data were obtained from 20 participants: 10 with normal cognitive function and 10 with cognitive impairment.

**Inclusion criteria:**

(1) Aged between 60 and 85 years (inclusive), regardless of gender;
(2) At least primary school education level, with the cognitive ability to complete all required assessments;
(3) Mandarin is the first or dominant language;
(4) Normal verbal communication, with pure-tone hearing thresholds ≤ 25 dB HL at 500, 1000, and 2000 Hz in at least one ear.

**Exclusion criteria:**

(1) Poor physical condition, including auditory or visual impairments, that would hinder the completion of cognitive or other study-related tests;
(2) Inability to complete all study assessments;
(3) Red-green color blindness (daltonism);
(4) Presence of other conditions potentially affecting speech articulation, such as cerebrovascular accidents, multiple sclerosis, stuttering, or vocal cord nodules.

**Figure 1:**
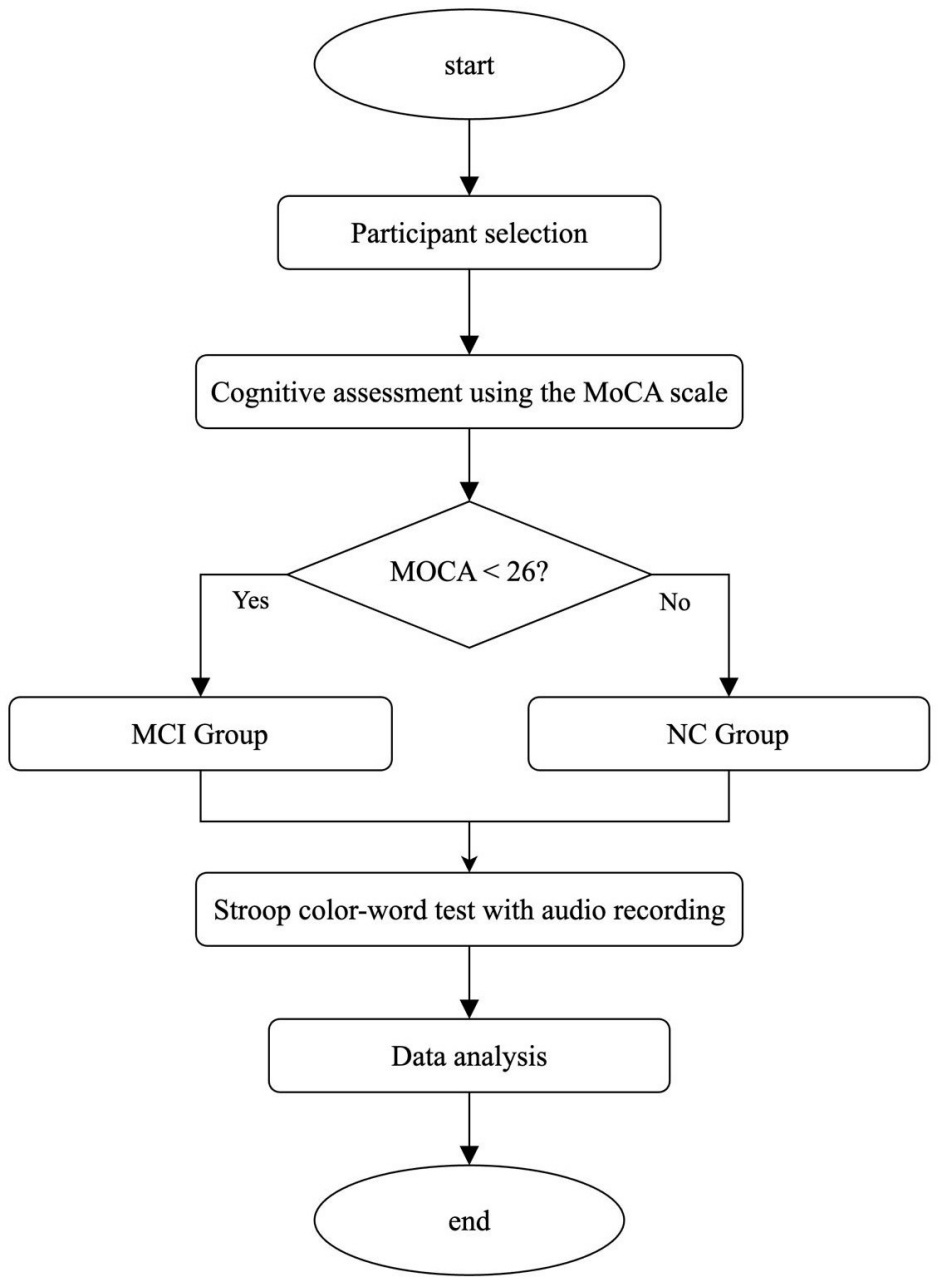
Experimental procedure diagram of determining MCI and NC based on MoCA scores.

All participants underwent cognitive screening using the Montreal Cognitive Assessment (MoCA), administered one-on-one in a quiet, controlled environment to minimize external interference. Prior to assessment, the study's purpose and procedures were thoroughly explained to each participant, including written assurances of personal information confidentiality. Participants were encouraged to ask questions, and efforts were made to reduce anxiety and ensure a testing environment conducive to optimal cognitive performance. MoCA administrators were trained to follow standardized scoring protocols with consistency and patience. Following cognitive evaluation, participants were stratified into groups based on their MoCA scores. Participants diagnosed with cognitive impairment met the 2011 National Institute on Aging and Alzheimer's Association (NIA-AA) diagnostic criteria. MoCA scores were used to classify cognitive impairment severity: mild cognitive impairment (scores 18–26). Cognitively normal participants met general eligibility criteria and achieved MoCA scores ≥ 26. The experimental procedure is outlined in Figure 1, and detailed participant demographics with MoCA scores are presented in Table 1.

**Table 1:** Participant information.

**NC group**				**MCI group**			
**MoCA**	**Education level**	**Age**	**Sex**	**MoCA**	**Education level**	**Age**	**Sex**
27	Senior secondary	74	Male	19	Junior secondary	83	Male
27	Senior secondary	71	Female	24	Junior college	65	Female
27	Undergraduate	85	Male	21	Senior secondary	61	Male
30	Undergraduate	72	Male	20	Senior secondary	67	Male
28	Undergraduate	69	Female	20	Junior secondary	81	Female
27	Technical secondary	71	Female	25	Undergraduate	73	Female
28	Postgraduate	66	Male	22	Junior secondary	71	Male
28	Undergraduate	80	Male	25	Primary	80	Male
27	Junior college	61	Male	24	Undergraduate	74	Male
27	Undergraduate	75	Female	24	Senior secondary	69	Female

### 2.2 Recording equipment

All participants in the MCI and NC (cognitively normal) groups completed the Stroop Color-Word Test, during which their verbal responses were digitally recorded for subsequent analysis of reaction time and accuracy. Speech samples were collected from 10 older adults with mild cognitive impairment (MCI) and 10 cognitively normal older adults using E-Prime software while performing the Stroop task. Extracted data included audio recordings, accuracy rates, and reaction time measurements triggered by Stroop stimuli. Speech samples were acquired in a controlled quiet environment (ambient noise ≤ 20 dB HL) following standardized recording protocols. Participants were seated upright, with their lips positioned 5-10 cm from the microphone, and instructed to provide natural speech responses. Verbal responses were recorded using E-Prime 3.0 experimental software, and audio signals were processed with Cool Edit Pro 2.1 for subsequent acoustic analysis.

**Figure 2:**
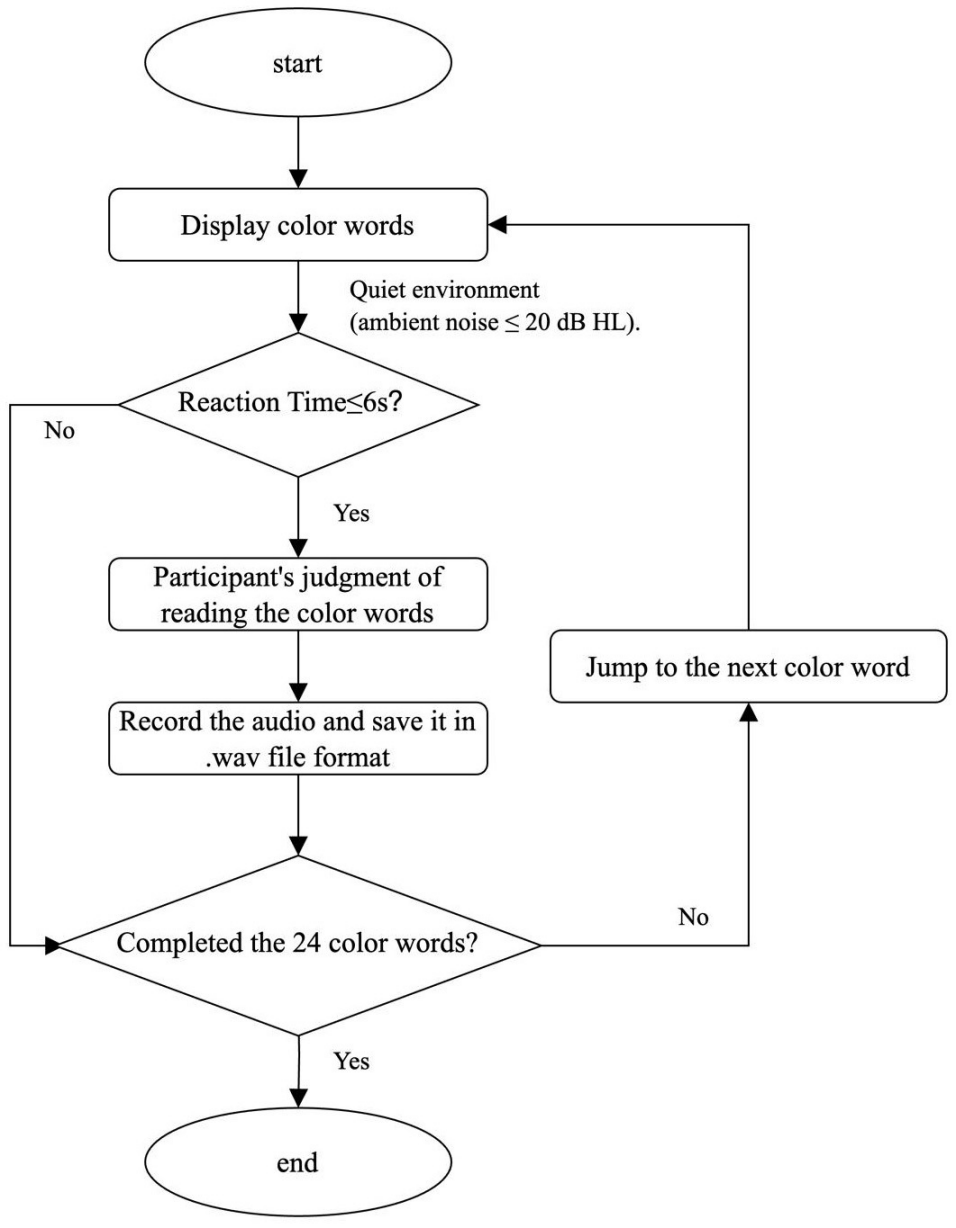
Flowchart of the E-Prime program design for the Stroop Color-Word Test.

**(1) E-Prime:** E-Prime, a comprehensive software suite for computerized behavioral experimentation, is jointly developed by Carnegie Mellon University, the University of Pittsburgh's Learning Research and Development Center, and Psychology Software Tools, Inc. Built on the E-Basic scripting language–syntactically similar to Visual Basic–it integrates experiment design, millisecond-precise stimulus presentation, and data collection within a unified graphical interface. Widely used across disciplines such as perception, memory, attention, psychophysiology, psycholinguistics, engineering psychology, developmental psychology, social psychology, and cognitive neuroscience, E-Prime (Kim et al., 2019) is optimized for behavioral experiments requiring high stimulus timing accuracy and flexibility. The platform supports multi-modal stimulus presentation (textual, visual, auditory, and combined modalities) and accommodates diverse response inputs, including keyboards, mice, response boxes (RBox), voice recording, and external devices. In this study, E-Prime was utilized to administer the Stroop Color-Word Task, present stimuli, record vocal responses, and save audio data in .wav format. The E-Prime program flowchart for the Stroop Color-Word Test procedure is depicted in Figure 2. The experimental design was implemented in E-Prime with standardized protocols for stimulus delivery, response capture, and timing measurements.

**(2) Cool Edit Pro 2.1:** Cool Edit Pro 2.1, a multi-track audio recording and editing software developed by Adobe Systems (El-Hadad and Brodie, 2019), provides powerful yet user-friendly tools for audio file processing. In this study, the software was used to isolate and edit single-channel speech signals meeting experimental criteria, storing them as .wav files with a 44.1 kHz sampling rate and 24-bit resolution. The software visualizes raw audio signals as time-domain waveforms, enabling highly accurate measurement of temporal parameters. Reaction times were quantified with millisecond precision, ensuring the reliability of timing data for subsequent analysis. Cool Edit Pro was employed to verify basic audio metadata, including sampling rate and number of channels. During the cropping stage, trial-specific audio segments were extracted using two methods: 1) manual segmentation via start/end markers based on E-Prime's behavioral data timestamps, or 2) automated batch processing for efficient segmentation. In the audio processing phase, noise reduction was achieved by capturing ambient noise samples, vocal clarity was enhanced using a parametric equalizer, volume consistency was standardized via normalization, and speech endpoints were detected precisely through spectrogram analysis. Post-processing, single-trial audio files were exported following uniform naming conventions to ensure accurate alignment with trial numbers, stimulus types, and other E-Prime behavioral data parameters.

### 2.3 Experimental procedure

Cognition refers to the mental processes of acquiring, storing, and applying knowledge or processing information, encompassing fundamental functions such as sensation, perception, memory, reasoning, imagination, and language. The Stroop test, a widely used cognitive assessment paradigm, exists in multiple variants. This study employed the commonly used color-word version, as visual interference typically outweighs auditory interference, with visual inputs often dominating cognitive processing–key reasons for selecting a visually based Stroop paradigm. In the Stroop paradigm, stimuli concurrently convey two types of information: the semantic meaning of a word and the color in which the word is printed. These dimensions are processed via distinct cognitive pathways, creating inherent conflict. When incongruent stimuli (e.g., the word “red” printed in blue ink) are presented, participants face challenges in selectively attending to one dimension while suppressing the other. Generally, response latency is shorter for word reading (a dominant automatic process) than for color naming (a non-dominant controlled process). Incongruent trials consistently elicit slower reaction times and higher error rates, reflecting the cognitive demand of interference suppression.

**Figure 3:**
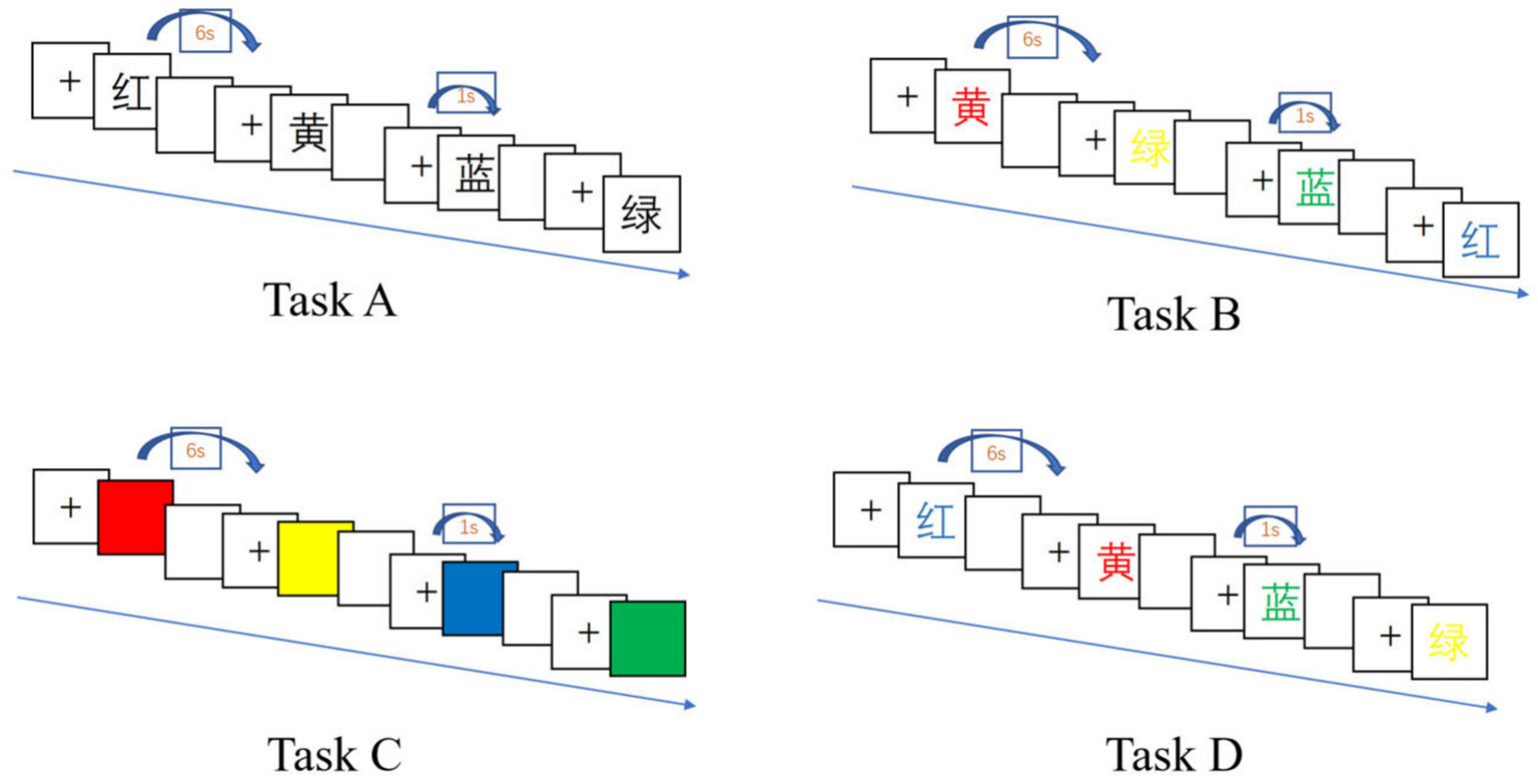
four tasks of Stroop Color-Word Test.

The present study employed the classic Stroop Color-Word Test paradigm (Scarpina and Tagini, 2017), consisting of four task types (A, B, C, D). Each task set included 24 trials across four color conditions (red, yellow, blue, green), with each color presented six times in randomized order. All tasks were administered via E-Prime software, which recorded participants' vocal responses as .wav files. Examples of the four task types are illustrated in Figure 3.

**Task A (word reading–neutral condition):** as depicted in Figure 3, participants were instructed to sit upright and maintain visual focus on the screen. When a Chinese character (black text on a white background) appeared, they were directed to read it aloud into the microphone with maximum speed and accuracy. A trained examiner positioned nearby verified response correctness in real time: pressing “1” for accurate pronunciations and “2” for errors. Regardless of accuracy, the system automatically advanced to the next stimulus either upon response confirmation or after a 6-second timeout. Trials exceeding the 6-second threshold were automatically coded as incorrect. All verbal responses were fully recorded by E-Prime for subsequent analysis.

**Task B (word reading—incongruent color background):** participants were instructed to read aloud the Chinese character presented in color (incongruent combinations of word meaning and font color, presented randomly). All other procedures mirrored those of Task A.

**Task C (color naming—neutral condition):** participants were required to name the color displayed on the screen (not in the form of a character) as quickly and accurately as possible. The operation procedure was identical to that of Task A.

**Task D (color naming—incongruent word stimulus):** participants were asked to name the color of the font in which a Chinese character was presented, where the character's semantic meaning and its font color were intentionally mismatched and randomized. All operational procedures followed those of Task A.

All statistical analyses were conducted using Statistical Package for the Social Sciences (SPSS) version 13.0. It is a professional statistical analysis software. The basic principle is based on statistical theory and data modeling, and its core goal is to reveal the patterns, relationships and trends in the data through quantitative methods. Levene method homogeneity test and multiple comparisons were performed to examine within-group differences in reaction times under various task conditions for both NC group and MCI group. A significance level of *p* < 0.05 was considered statistically significant. Independent-samples t-tests were used to compare between-group differences (NC vs. MCI) in reaction times and accuracy rates under the same task conditions. Statistical significance was defined as *p* < 0.05.

## 3 Results

### 3.1 Response accuracy in the Stroop Color-Word Task

The accuracy scores and number of correct responses for the Stroop color-word tasks were compared between NC group and MCI group. The results are summarized in Table 2. For each task (i.e., A, B, C, and D), each subject underwent 24 experiments, and the number of correct responses taken within the prescribed time was recorded. Statistical analysis using SPSS indicated that there were no significant differences between the NC and MCI groups in terms of the number of correct responses for Task Types A, B, and C (p ≥ 0.05). However, for Task D and the overall accuracy rate, the differences between groups were statistically significant (*p* < 0.05). Detailed results of the statistical analysis are presented in Table 3.

**Table 2:** Response number of Stroop Color-Word Test Task for the NC group and MCI group.

**NC group**					**MCI group**				
**A**	**B**	**C**	**D**	**Accuracy**	**A**	**B**	**C**	**D**	**Accuracy**
24.00	24.00	24.00	24.00	100.00%	24.00	24.00	24.00	24.00	100.00%
24.00	24.00	24.00	24.00	100.00%	24.00	24.00	24.00	23.00	98.96%
24.00	24.00	24.00	24.00	100.00%	24.00	24.00	24.00	23.00	98.96%
24.00	24.00	24.00	24.00	100.00%	24.00	24.00	24.00	22.00	97.92%
24.00	24.00	24.00	23.00	98.96%	24.00	24.00	22.00	21.00	94.79%
24.00	24.00	24.00	24.00	100.00%	24.00	24.00	24.00	23.00	98.96%
24.00	24.00	24.00	24.00	100.00%	24.00	24.00	24.00	22.00	97.92%
24.00	24.00	24.00	21.00	96.88%	24.00	24.00	24.00	22.00	97.92%
24.00	24.00	24.00	22.00	97.92%	24.00	24.00	24.00	21.00	95.83%
24.00	24.00	24.00	24.00	100.00%	24.00	24.00	24.00	23.00	98.96%

**Table 3:** Response accuracy of Stroop Color-Word Test Task for the NC group and MCI group.

	**NC**	**MCI**	***t* value**	***p* value**
Task A	24.00 ± 0.00	24.00 ± 0.00	-	-
Task B	24.00 ± 0.00	24.00 ± 0.00	-	-
Task C	24.00 ± 0.00	23.80 ± 0.63	1.000	0.331
Task D	23.40 ± 1.07	22.40 ± 0.97	2.188	0.042^*^
Accuracy(%)	99.38 ± 1.12	98.02 ± 1.59	2.204	0.041^*^

^*^p < 0.05.

### 3.2 Reaction times comparison in the Stroop Color-Word Test Task

The reaction time data for Task A, B, C, and D in the Stroop color-word test for both the NC and MCI groups are presented in Tables 4 and 5, respectively.

**Table 4:** Reaction times (*ms*) of Task A and B for the NC group and MCI group.

	**Task A**				**Task B**			
	**Mean**	**Median**	**Maximum**	**Minimum**	**Mean**	**Median**	**Maximum**	**Minimum**
NC	624.71	618.50	810.00	554.00	688.75	681.00	835.00	583.00
	563.25	528.50	876.00	421.00	604.83	580.00	848.00	414.00
	604.21	604.50	742.00	530.00	746.88	740.00	1,045.00	600.00
	497.54	490.00	614.00	413.00	538.92	541.00	648.00	430.00
	407.26	405.00	477.00	365.00	432.67	421.50	537.00	359.00
	523.13	513.00	670.00	454.00	552.17	529.00	748.00	463.00
	566.17	556.00	741.00	459.00	720.83	725.00	960.00	499.00
	663.25	650.00	1,119.00	495.00	720.46	695.00	1,080.00	505.00
	780.88	727.00	1,396.00	605.00	721.13	707.00	958.00	553.00
	479.43	478.00	551.00	411.00	601.67	604.00	855.00	461.00
MCI	732.00	679.50	1,127.00	543.00	778.23	708.00	1,129.00	597.00
	782.91	718.00	1,782.00	566.00	987.63	903.50	1,825.00	580.00
	890.63	709.00	1,947.00	586.00	667.96	649.50	852.00	533.00
	679.54	665.00	961.00	527.00	966.88	923.50	1,518.00	687.00
	762.29	747.50	961.00	591.00	701.48	691.00	795.00	607.00
	773.70	774.00	947.00	649.00	886.08	847.00	1,158.00	670.00
	550.04	539.00	743.00	430.00	733.83	619.00	1,284.00	426.00
	733.00	737.00	867.00	517.00	847.42	740.50	1,408.00	666.00
	572.54	527.50	1,082.00	399.00	577.17	566.50	825.00	394.00
	656.83	622.50	879.00	527.00	698.91	666.00	964.00	479.00

**Table 5:** Reaction times (*ms*) of Task C and D for the NC group and MCI group.

	**Task C**				**Task D**			
	**Mean**	**Median**	**Maximum**	**Minimum**	**Mean**	**Median**	**Maximum**	**Minimum**
NC	737.22	727.00	892.00	594.00	752.58	720.50	1273.00	594.00
	683.43	672.00	989.00	501.00	810.63	785.00	1,268.00	550.00
	741.50	733.00	1,282.00	514.00	1,109.20	1,024.50	2,089.00	604.00
	574.58	568.00	717.00	426.00	1,088.26	1,052.00	2,411.00	764.00
	574.41	553.50	947.00	366.00	1,099.23	906.50	2,373.00	617.00
	643.92	612.00	1,078.00	496.00	978.09	935.00	1,528.00	697.00
	755.30	760.00	1,068.00	510.00	898.92	850.00	1,452.00	583.00
	897.25	680.50	2,647.00	490.00	768.21	808.00	1,600.00	672.00
	1,051.46	915.00	3,260.00	739.00	651.13	638.50	964.00	503.00
	582.52	594.00	773.00	437.00	1,024.88	774.00	2,229.00	618.00
MCI	992.79	985.00	1,285.00	747.00	1,156.45	1,086.50	2,394.00	855.00
	975.92	928.00	1,761.00	649.00	1,240.45	1,193.50	1,809.00	837.00
	891.42	889.50	1,265.00	711.00	1,951.83	1,932.50	3,839.00	1,014.00
	838.17	843.00	1,037.00	551.00	1,307.80	1,321.00	2254.00	818.00
	860.79	798.50	1,700.00	507.00	1,653.50	1,444.50	2,754.00	897.00
	1,224.71	1,130.00	2,660.00	761.00	1,401.88	1,189.00	2,250.00	836.00
	1,235.57	1,195.00	2,120.00	598.00	1,297.25	1,145.50	2,381.00	1,617.00
	1,191.70	1,169.00	2,243.00	477.00	1,765.33	1,618.00	3,206.00	991.00
	1,187.33	1,129.00	2,080.00	836.00	1,083.83	909.00	2,717.00	633.00
	804.52	806.00	974.00	617.00	1,405.85	1,327.00	2,874.00	889.00

The variance analysis and effect size estimation of the reaction time under different task states in the NC group and the MCI group were conducted respectively using SPSS software. The results showed that the influence of different task states on the reaction time was statistically significant, in the NC group (*P* = 0.000, η^2^ = 0.637) and the MCI group (*P* = 0.000, η^2^ = 0.721). The Levene method homogeneity test showed that the reaction times of each task in the NC group were homogeneous in variance (*F* = 2.124, *P* = 0.114), while the reaction times of each task in the MCI group were not homogeneous in variance (*F* = 3.781, *P* = 0.019). The NC group used the least significant difference (LSD) test for multiple comparisons among various tasks, while the MCI group used the Tamhane's T2 test for multiple comparisons among various tasks. The results all showed that there were differences in the reaction time between Task A and task C (*P* < 0.05). There was A significant difference in the reaction time between task D and tasks A, B, and C (*P* < 0.01). Detailed results of the group comparison are provided in Table 6.

**Table 6:** Comparisons of mean reaction time differences between different task conditions.

	**NC group**		**MCI group**	
	**Reaction time difference**	***p*** **value**	**Reaction time difference**	***p*** **value**
A and B	−61.848	0.320	−71.211	0.737
A and C	−153.176	0.017^*^	−204.765	0.027^*^
A and D	−449.304	0.000^**^	−713.069	0.000^**^
B and C	−91.328	0.145	−133.554	0.326
B and D	−387.461	0.000^**^	−641.858	0.000^**^
C and D	−296.133	0.000^**^	−508.304	0.001^**^

^*^p < 0.05, ^**^p < 0.01.

Independent-samples t-tests were conducted using SPSS 13.0 to compare the reaction times of the NC and MCI groups under each of the four Stroop task conditions (Tasks A, B, C, and D). Significant differences were found between the two groups in both the mean reaction times across all task types (*p* < 0.05), indicating that the MCI group generally required longer processing time than the NC group (as shown in Table 7).

**Table 7:** Comparison of mean reaction time between the NC group and MCI group (*mean* ± *std*).

	**NC(*ms*)**	**MCI(*ms*)**	***t* value**	***p* value**
Task A	570.983 ± 105.153	713.348 ± 102.129	−3.07	0.007^**^
Task B	632.831 ± 103.547	784.559 ± 134.244	−2.830	0.011^*^
Task C	724.159 ± 153.291	918.113 ± 165.441	−2.719	0.014^*^
Task D	1020.292 ± 173.177	1426.417 ± 278.707	−3.914	0.001^**^

^*^p < 0.05, ^**^p < 0.01.

### 3.3 Correlation analysis of MoCA scores with accuracy rate and reaction time

**Figure 4:**
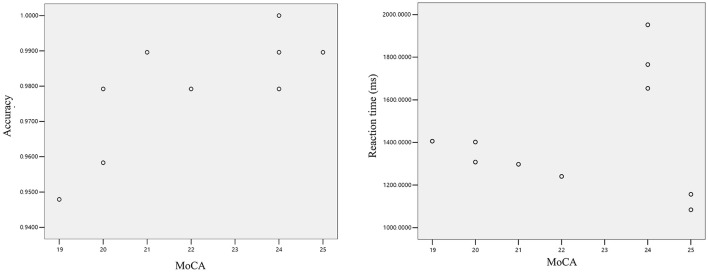
The relationship between the MoCA score and the reaction accuracy and reaction time.

In cognitive neuroscience, investigating the associations between cognitive function metrics in mild cognitive impairment (MCI) patients is critical for early disease diagnosis and intervention. This section employs Pearson correlation analysis to determine whether linear relationships exist between Montreal Cognitive Assessment (MoCA) scores and both accuracy rates and reaction times during the interference suppression task (Task D) in MCI patients. As illustrated in Figure 4, the scatter plot demonstrates a distinct positive trend in data point distribution, visually supporting a close relationship between MoCA scores and reaction accuracy. Statistical analysis revealed a significant positive linear correlation between MoCA scores and accuracy rates in the MCI group (*r* = 0.758, *p* = 0.011), indicating that higher MoCA scores are associated with significantly improved task accuracy. This reflects a strong co-directional trend with robust statistical significance. Conversely, correlation analysis between MoCA scores and Task D reaction times showed no evidence of a linear relationship (*r* = 0.144, *p* = 0.691). Although no evidence of a correlation between the two was found, the scatter plot shows a trend that the higher the MoCA score, the less time required for the reaction. This situation may occur for the following reasons: (1) The overall sample size is relatively small, which cannot accurately reflect the overall trend. (2) There were situations such as inattentiveness or fatigue during the testing process, which led to excessive deviations in some samples.

## 4 Discussion

The Stroop Color-Word Test, a classic paradigm in cognitive psychology, is widely used in clinical research to assess cognitive function. In this study, the Stroop test served as the experimental model to investigate cognitive differences between MCI patients and cognitively normal older adults, with a focus on reaction accuracy and reaction times. Data were collected from 10 cognitively normal older adults and 10 MCI patients recruited from the First Affiliated Hospital and community of Jinan University. The experiment comprised four task types (A, B, C, D), each containing 24 trials (4 colors × 6 repetitions). Tasks A and C represented single-task conditions (word reading or color naming), whereas Tasks B and D involved dual-task conditions presenting concurrent semantic and color information, necessitating selective attention. Processing incongruent information imposes significant cognitive load: word reading reflects a dominant automatic response, while color naming constitutes a non-dominant controlled response. In Task D, participants were required to suppress the dominant semantic response in favor of color naming–a process termed interference inhibition.

Our findings revealed no significant between-group differences in accuracy for Tasks A, B, and C (p ≥ 0.05), but a statistically significant difference emerged for Task D and overall accuracy (*p* < 0.05). These results suggest that MCI patients exhibit higher error rates during tasks requiring robust cognitive control and interference suppression. In contrast, both groups demonstrated comparable performance on simpler tasks, aligning with the hypothesis that early-stage MCI spares basic cognitive processing while impairing performance under elevated cognitive load. MCI is typically characterized by early deficits in recent memory, with remote memory impairment emerging as the condition progresses. The Stroop task stimuli (e.g., Chinese characters for red, yellow, blue, green) represented highly familiar, everyday vocabulary, and participants–recruited from a relatively well-educated community–exhibited strong baseline recognition, minimizing errors in basic stimulus identification. This contextual familiarity likely explains the equivalent accuracy observed in Tasks A, B, and C across groups. Conversely, Task D, which demanded inhibition of the dominant semantic response, imposed a significant cognitive workload, leading to pronounced declines in processing speed and accuracy, particularly among MCI patients. This pattern underscores the vulnerability of interference control mechanisms in individuals with MCI, highlighting their reduced capacity to resolve cognitive conflict during dual-task demands.

These findings are supported by the theory of dominant vs. non-dominant response hierarchies. When presented with color-word stimuli, participants automatically process semantic content–a core feature of automatic semantic activation. Even when instructed to disregard word meaning, this information is involuntarily encoded, as evidenced by participants' tendency to mistakenly read the character instead of naming the font color in Task D, reflecting the dominance of automatic semantic processing. To characterize the speed disparity between dominant and non-dominant responses, Tasks A (word reading) and C (color naming) were compared. Both NC and MCI groups exhibited significant differences in mean and median reaction times (*p* < 0.05), with word reading (dominant response) reliably faster than color naming (non-dominant response). This aligns with the theoretical framework of automatic semantic priority, where familiar linguistic processing occurs with minimal cognitive effort. Comparing Task A (word reading) and Task B (reading words with incongruent font colors) revealed no between-group differences in mean reaction times (p ≥ 0.05), indicating that color interference did not prolong response latency for either group. This suggests that non-dominant color processing cannot effectively compete with dominant semantic processing, consistent with the brain's prioritization of automatic semantic pathways. These results reinforce the principle of automaticity in semantic dominance. Contrasting Tasks A and D highlighted a striking processing cost for interference suppression: both groups exhibited nearly double the reaction time in Task D compared to Task A. While Tasks B and D both involve dual information input, only Task D requires active suppression of the dominant response.

**Figure 5:**
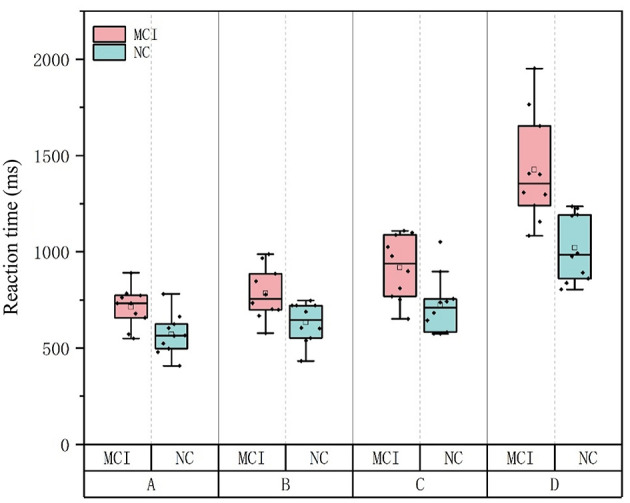
Mean reaction times of NC and MCI groups across various tasks.

The primary objective of this study was to investigate information processing differences between MCI patients and cognitively normal older adults, with a focus on early diagnostic markers and intervention targets. Independent-samples t-tests revealed statistically significant group differences between NC and MCI participants across all Stroop task types (A-D) (*p* < 0.05), demonstrating that MCI patients exhibit globally slower processing speeds (Figure 5). A positive linear correlation was observed between MoCA scores and task accuracy (*r* = 0.758, *P* = 0.011). This indicates that declining cognitive impairment is associated with both slower information processing and increased error rates during complex tasks. These findings align with prior research, such as Borella et al. (2017), which documented reduced resistance to proactive interference and impaired executive control in MCI. The observed deficits in interference suppression may serve as an early detectable marker of MCI. For example, Chehrehnegar et al. (2019) used eye-tracking to show that MCI and AD patients exhibit prolonged saccadic movements, with MCI individuals relying on extended processing time to maintain accuracy–a strategy that becomes increasingly inefficient as task difficulty escalates. This study was carried out based on the dual-process theoretical framework of “automatic semantic activation–inhibition control”, but functional neuroimaging data (such as fMRI) were not integrated to explore the neural mechanisms of the prefrontal limbic system, resulting in difficulties in precisely locating the brain regions corresponding to cognitive behavioral deficits. Subsequent studies will combine functional imaging data to construct a “behavioral index - neural matrix” correlation model to deepen the understanding of the relationship between cognitive behavior and neural basis.

## 5 Conclusion

This study demonstrates that dominant cognitive responses require less processing time, whereas tasks involving interference suppression elicit significantly longer reaction times and higher error rates. Compared to cognitively normal older adults, those with mild cognitive impairment (MCI) exhibited slower response speeds and heightened susceptibility to proactive interference. The MCI group showed more pronounced deficits in interference suppression, as evidenced by both prolonged reaction times and increased error rates. These findings suggest that compromised interference control mechanisms in MCI may serve as potential biomarkers for early detection of cognitive decline. The differential performance on Stroop tasks illuminates the nature of cognitive processing deficits in MCI, offering actionable insights for developing targeted interventions in early-stage impairment. The significant positive correlation between MoCA scores and task accuracy provides a foundation for using the MoCA to evaluate and predict cognitive task performance. Conversely, the lack of linear correlation between MoCA scores and Task D reaction times highlights potential limitations of the MoCA in assessing interference inhibition abilities in MCI, underscoring the need for comprehensive evaluation using targeted assessment metrics alongside traditional cognitive scales.

## Data Availability

The raw data supporting the conclusions of this article will be made available by the authors, without undue reservation.
